# Lycorine ameliorates liver steatosis, oxidative stress, ferroptosis and intestinal homeostasis imbalance in MASLD mice

**DOI:** 10.1186/s10020-024-01003-6

**Published:** 2024-11-27

**Authors:** Ziwen Wang, Mengpei Zhu, Qian Li, Jiali Cao, Qiangqiang Zhong, Ze Jin, Yumei Huang, Qing Lan, Ya Gao, Zhifan Xiong

**Affiliations:** 1 Department of Gastroenterology, Liyuan Hospital, Tongji Medical College, Huazhong University of Science and Technology, Wuhan, China; 2 Department of Integrated Traditional Chinese and Western Medicine, Liyuan Hospital, Tongji Medical College, Huazhong University of Science and Technology, Wuhan, China; 3 Present address: #39 Yanhu Avenue, East Lake Scenic Area, Wuhan, 430077 Hubei China

**Keywords:** Metabolic dysfunction-associated steatotic liver disease, Lycorine, Oxidative stress, Ferroptosis, Intestinal homeostasis

## Abstract

**Background:**

Metabolic dysfunction-associated steatotic liver disease (MASLD) is the most common liver disease worldwide and few drugs are available for its treatment. Lycorine has effective anti-inflammatory and lipid-lowering effects, but the impact on MASLD is not fully understood. In this study, we intend to test the intervention effect of lycorine on MASLD.

**Methods:**

A MASLD mouse model was constructed on a high-fat diet for 16 weeks, and low, medium, and high doses of lycorine were given by gavage for the last 4 weeks. Detecting indicators related to liver steatosis, oxidative stress, and ferroptosis. In vivo and in vitro experiments co-validate potential targets identified by network pharmacology, molecular docking and western blot for lycorine intervention in MASLD liver. A combination of pathology, western blot, qRT-PCR, and 16 S rRNA sequencing verified adipose tissue and intestinal alterations.

**Results:**

Lycorine ameliorated hepatic steatosis, oxidative stress and ferroptosis in MASLD mice by inhibiting the expression of phosphorylated EGFR, inhibiting the PI3K/AKT signaling pathway. We also observed a dose-dependent effect of lycorine to improve some of the indicators of MASLD. In vitro, knockdown of EGFR significantly attenuated palmitic acid-induced hepatocyte steatosis. In addition, lycorine promoted WAT browning for thermogenesis and energy consumption, affected the composition of intestinal flora, improved the intestinal barrier, and reduced intestinal inflammation.

**Conclusions:**

EGFR was the target of lycorine intervention in MASLD. Lycorine ameliorated hepatic steatosis, oxidative stress and ferroptosis by affecting the EGFR/PI3K/AKT signaling pathway in MASLD mice. Furthermore, lycorine promoted WAT browning and ameliorated intestinal homeostatic imbalance. The above effects may also have dose-dependent effects.

**Supplementary Information:**

The online version contains supplementary material available at 10.1186/s10020-024-01003-6.

## Background

The incidence of metabolic dysfunction-associated steatotic liver disease (MASLD) is increasing year by year and it is now the most common chronic liver disease in the world (Miao et al. 2024). MASLD can induce a wide range of extrahepatic manifestations such as cardiovascular disease, chronic kidney disease and certain extrahepatic cancers. Once MASLD progresses to the end stage of liver disease, therapeutic outcomes are limited and liver transplantation may be required. Nowadays, metabolic dysfunction-associated steatohepatitis has become the leading cause of liver transplantation, which imposes a huge economic burden and poses a major challenge to the global economy (Paklar et al. 2023). Therefore, early detection and intervention of MASLD is crucial.

Studies have shown that multiple factors are involved in the pathogenesis of MASLD, the most important of which appear to be genetic factors, nutritional habits, insulin resistance, adipogenesis, gut microbiota, inflammation, oxidative stress (OS) and mitochondrial/metabolic remodeling (Karkucinska-Wieckowska et al. 2022). OS plays a key role in the onset and progression of MASLD, which is characterized by a pro-oxidant imbalance between reactive oxygen species (ROS) production and antioxidants (Spahis et al. 2017). This imbalance can lead to disturbances in signaling and redox homeostasis and/or damage to various cellular structures, affecting their function and contributing to the development of a variety of chronic diseases. Ferroptosis is a novel form of programmed cell death driven by inactivation of the lipid repair enzyme glutathione peroxidase 4 (GPX4) and subsequent accumulation of iron-dependent lipid peroxides (Yang and Stockwell 2016). OS is closely related to ferroptosis due to similar pathological processes, and both play an important role in the development of MASLD (Zhu et al. 2023).

Few drugs are currently approved for the treatment of MASLD. Lycorine, an alkaloid present in the bulbs of the stonecrop plant, Allium sativum, has potent anti-inflammatory, antiviral, and antitumor activities, but the effects on MASLD are not fully understood (Liang et al. 2020). It was confirmed that lycorine significantly reduced lipid levels in serum and tissues of mice (Zheng et al. 2021) as well as OS in the liver of rats with hepatic fibrosis (Alkreathy and Esmat 2022). Therefore, we hypothesized that lycorine may be a potential drug for the treatment of MASLD. In the present study, we constructed a mouse model of MASLD by a 16-week high-fat diet (HFD) and set up a lycorine drug concentration gradient to investigate the effects and potential mechanisms of lycorine gavage for 4 weeks in MASLD mice. In addition, we performed further in vitro experiments to verify the molecular signaling of lycorine using small interfering RNA (siRNA).

## Materials and methods

### MASLD mouse models

**Fig. 1:**
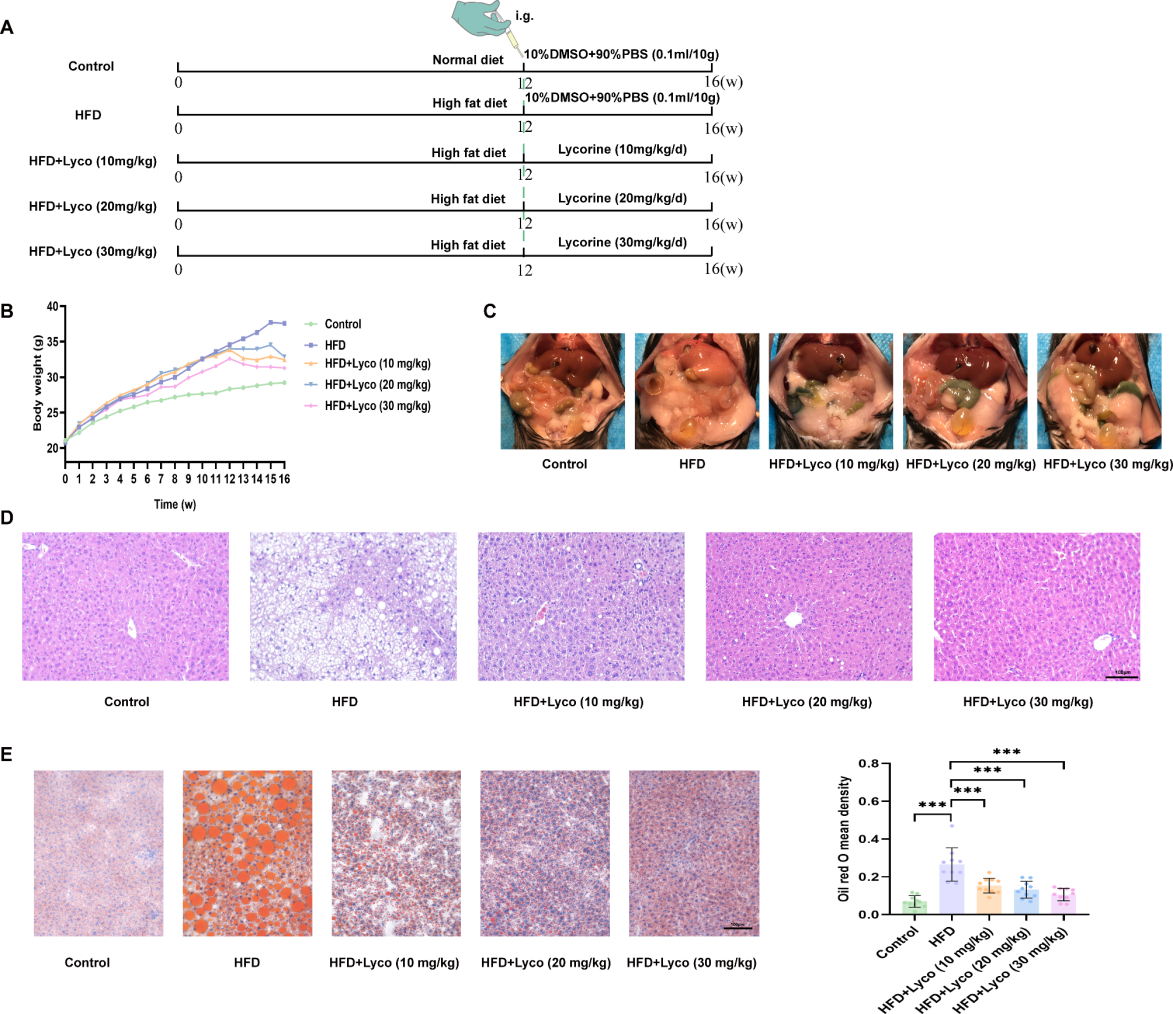
Lycorine reduced body weight and ameliorated hepatic pathological changes in MASLD mice. (**A**) schematic diagram of mouse modeling and intervention; (**B**) body weight changes in each group of mice; (**C**) naked eye view of abdominal dissection; (**D**) HE staining of livers; (**E**) oil red O staining of livers and semi-quantitative analysis (*n* = 10). Scale bar = 100 μm. ^***^P < 0.001 vs. HFD

Fifty six-week-old male C57BL/6J mice (weighing 18–20 g) were purchased from Hunan Slake Jingda Experimental Animal Co., Ltd. After adaptive feeding for 1 week, they were randomly divided into 5 groups on average. The normal group was given a normal diet, while the HFD group and lycoline low, medium, and high dose group were given a HFD (D12492, 60% of energy comes from fat) (ReadyDietech, China). Among them, mice in the low, medium, and high dose group of lycorine (Shanghai yuanye Bio-Technology Co., Ltd, China) (10 or 20 or 30 mg/kg body weight) were given daily gavage of lycorine, while normal and HFD groups were given PBS by gavage in the last four weeks (Fig. 1A). All mice were given a gavage of 0.1mL/10 g. On the 16th weekend, mice were euthanized after isoflurane anesthesia. This animal experiment has been approved by the Animal Ethics Committee of Tongji Medical College, Huazhong University of Science and Technology, and complies with the rules of animal experiments.

### Hematoxylin and eosin (HE) staining

Fresh tissues were fixed in 10% formalin for 24 h. After dehydration, transparency, and embedding into wax blocks, the tissues were cut into 4 μm thick slices. Sections were baked at 60℃ for 1 h, then dewaxed, stained, dehydrated, transparent, sealed with neutral gum and photographed under a light microscope.

### Oil red O staining

Fresh liver tissues were embedded in OCT embedding agent, and 10-µm-thick sections were cut by a frozen sectioning machine. Stain according to the instruction (NJJCBIO, China).Five fields of view of each mouse liver section were randomly selected, and Image-Pro Plus 6.0 calculated the ratio of the area of lipid droplets in each section to the total area of the picture and then took the average value for semi-quantitative analysis.

### Transmission electron microscope (TEM)

Fresh liver tissue (2 × 2 × 2 mm^3^) was fixed in 2.5% glutaraldehyde and stored at 4℃. Ultrathin Sect. (50–100 nm) were prepared by fixing with osmium acid at 4 ℃ for 4 h, gradient dehydration with acetone at room temperature (70% acetone for 10 min, 90% acetone for 10 min, 100% acetone I for 10 min, 100% acetone II for 10 min), embedding, and oven curing (overnight at 85 ℃). After double staining with uranium acetate lead citrate, they were observed and photographed (Hitachi). The morphological changes of mitochondria, including size and ultrastructure, were observed.

### The indicators of liver function in serum and liver tissue

At the end of the 16th week, blood was taken from the eyeballs and left at room temperature for 1 h. Serum was separated by centrifugation at 3500r/min, 4℃ for 15 min. Serum and liver ALT, AST, TG, TC, and serum HDL-c, LDL-c were measured within 1 week according to the kit instructions (NJJCBIO, China).

### Liver OS indicators and tissue iron and ferrous iron (Fe^2+^) content determination

Liver tissue was weighed and 10% liver homogenate was prepared. The reagents were added in sequence according to the instructions for the determination of catalase (CAT), glutathione (GSH), glutathione peroxidase (GSH-Px), malondialdehyde (MDA), superoxide dismutase (SOD), tissue iron content (NJJCBIO, China) and Fe^2+^ content (Beijing Solarbio Science & Technology Co., Ltd., China), respectively. Then reagents were added sequentially and read by enzyme marker. The details of the above experimental methods were shown in the Supplementary file. Protein quantification was performed by Bradford kit (Sevenbio, Beijing, China).

### ROS detection

Take 50 mg of liver tissue and add 1mL PBS to prepare tissue homogenate. After centrifugation at 100 g for 5 min at 4℃, 200µL of supernatant was added into a black 96-well plate, and then 0.5µL of DHE probe was added, and mixed thoroughly. The fluorescence intensity was detected by fluorescence zymography (excitation wavelength of 535 nm, emission wavelength of 610 nm) after incubation at 37℃ and protected from light for 30 min. 20 µL of diluted homogenate was taken for protein quantification using Bradford kit. ROS intensity = fluorescence intensity (RFU)/protein concentration (mgprot).

### qRT-PCR

Total RNA was isolated by FreeZol Reagent (Vazyme Biotech Co., Ltd, China), then the RNA was reverse transcribed to cDNA. 4.6 µL of diluted cDNA, 0.4 µL of diluted primers and 5 µL of 2× SYBR Green qPCR MasterMix II (Sevenbio, China) were added for quantitative fluorescence analysis. The primers are displayed in Table 1.

**Table 1:** Primers for qRT-PCR

Gene name	Primer Sequence (5′ → 3′)
SREBP-1	Forward: GGATCGCAGTCTGAGGAGGAG Reverse: CCAGGAGCCGACAGGAAGG
SCD1	Forward: TTCCGCCACTCGCCTACAC Reverse: GATAGTCAGTTGCTCGCCTCAC
CD36	Forward: AGGAGTGCTGGATTAGTGGTTAGG Reverse: CAGGAGAGGCGGGCATAGTATC
PPAR-α	Forward: CACTTGCTCACTACTGTCCTTGG Reverse: TGCTGGTATCGGCTCAATAATTCC
PPAR-γ	Forward: TGTTCGCCAAGGTGCTCCAG Reverse: AGGCTCATGTCTGTCTCTGTCTTC
FASN	Forward: TCCTGAAGCCGAACACCTCTG Reverse: GCGACAATATCCACTCCCTGAATC
HSL	Forward: AGAGACACCAGCCAACGGATAC Reverse: TTGCGGTTAGAAGCCACATAGC
HMGCR	Forward: GCAGATGCTAGGTGTTCAAGGAG Reverse: CAGCCATCACAGTGCCACATAC
CPT-1α	Forward: CACAACAACGGCAGAGCAGAG Reverse: ACACCACATAGAGGCAGAAGAGG
CCL2	Forward: CCACAACCACCTCAAGCACTTC Reverse: AATTAAGGCATCACAGTCCGAGTC
CCL5	Forward: CGAAGGAACCGCCAAGTGTG Reverse: CTAGGACTAGAGCAAGCAATGACAG
CXCL10	Forward: CAGGCTCGTCAGTTCTAAGTTTACC Reverse: CCTTGGGAAGATGGTGGTTAAGTTC
UCP1	Forward: GGAGGTGTGGCAGTGTTCATTG Reverse: GCTTTCTGTGGTGGCTATAACTCTG
PGC-1α	Forward: TCGCTGCTCTTGAGAATGGATATAC Reverse: TCGTCTGAGTTGGTATCTAGGTCTG
Prdm16	Forward: GCCGTTCAAGTGCCATCTGTG Reverse: CCTCGTGTTCGTGCTTCTTCAG
Cidea	Forward: CCGTGTTAAGGAATCTGCTGAGG Reverse: GGATGGCTGCTCTTCTGTATCG
β-actin	Forward: ACTGCCGCATCCTCTTCCTC Reverse: AACCGCTCGTTGCCAATAGTG

### Network Pharmacology

#### Identification of lycorine-related targets

The Canonical SMILES structure of lycorine was obtained from the PubChem database (https://pubchem.ncbi.nlm.nih.gov*)*, and its related targets were obtained by importing the Canonical SMILES structure of lycorine in the SwissTarget Prediction database (http://www.swisstargetprediction.ch*).*

#### The selection of MASLD disease-associated targets

The keyword “non-alcoholic fatty liver disease” was used as a qualifier in the GeneCards (https://www.genecards.org/*)*, OMIM (http://www.omim.org/*)*, DisGeNET (https://www.disgenet.org/*)* (Pinero et al. 2021), TTD (https://db.idrblab.net/ttd/*)* (Zhou et al. 2024) and Drugbank (https://go.drugbank.com/*).* The restriction was “Homo sapiens” to screen the ensemble of potential targets for MASLD.

#### Protein-protein interaction (PPI) network construction and core target screening

An online tool (http://bioinformatics.psb.ugent.be/webtools/Venn/*)* was used to intersect the lycorine-related targets with the ensemble of MASLD disease targets and to draw a Venn diagram. The intersected genes were then transferred to the STRING database platform (https://cn.string-db.org/*)* to construct a PPI network model, and the biological species “Homo sapiens” was selected for searching, and the scoring condition was set to “highest confidence>0.4”. Cytoscape 3.9.1 software was used for visualization, and the topological parameters of Degree, Betweenness, and Closeness were analyzed by the CentiScaPe 2.2 plug-in to assess the central attributes of the nodes in the network, and ultimately to obtain the core protein interaction network.

The ferroptosis-related suppressor and driver were downloaded from the FerrDb website (http://www.zhounan.org/ferrdb/current/*)* (Zhou et al. 2023) and plotted against potential targets of MASLD and targets of lycorine action in a Venn diagram. The three intersecting genes were used to construct a PPI network, and the high-scoring targets were selected for molecular docking.

#### Molecular docking

Search the 3D structure of the target in the RCSB PDB (https://www.rcsb.org/*)* database and download the 3D structure with Resolution value < 2 A. The smaller the Resolution value, the higher the resolution. And the proteins were dehydrogenated, ligand cleared and hydrogenated using Pymol and AutoDock tools, and then molecular docking was performed using AutoDock tools, and visualized using Pymol software after docking.

#### **KEGG signaling pathway analysis**

The intersecting targets of MASLD and lycorine were imported into the online analysis software Sangerbox (http://sangerbox.com/home.html*)* (Shen et al. 2022).

### ELISA

Epidermal growth factor receptor (EGFR) Assay Kit was bought from Bioswamp (MU30453). The reagents were added sequentially according to the instructions and the absorbance was detected at 450 nm.

### Western blot

Mouse tissues were weighed separately and protein samples were prepared by lysis, centrifugation, and boiling after addition of loading buffer. Proteins were separated by 10% or 12% SDS-PAGE and subsequently transferred to PVDF membrane. 5% skimmed milk powder was blocked, washed with TBST, and the primary antibody was incubated in a shaker overnight at 4 °C. After washing with TBST, the HRP-conjugated secondary antibody was incubated at room temperature for 1 h. After washing with TBST again, the antibody was developed with the ultra-sensitive ECL luminescent solution (New Cell & Molecular Biotech, China). Image J was used for quantitative protein analysis. The primary antibodies were purchased from Nature Biosciences, Wanleibio, Abmart, CST, Proteintech, UpingBio, Abclonal, MedChemExpress (MCE) and Boster, separately. The detailed information of primary antibodies was shown in Table 2.

**Table 2:** The detailed information of primary antibodies

Name	Company	Cat No	Dilution
anti-phospho-EGFR (Y1068)	Nature Biosciences	A11041	1:1000
anti-EGFR	Wanleibio	WL0682a	1:1000
anti-PI3-kinase p85-alpha/gamma (Tyr467/199)	Abmart	T40116	1:1000
anti-PI3 kinase p85	CST	4292	1:1000
anti-phospho-AKT (Ser473)	Wanleibio	WLP001a	1:1000
anti-AKT	Proteintech	80816-1-RR	1:5000
anti-p62	Nature Biosciences	A95292	1:1000
anti-Keap1	MCE	HY-P80732	1:1000
anti-Nrf2	Abmart	T55136	1:1000
anti-HO-1	Abmart	T55113	1:1000
anti-NQO1	Nature Biosciences	A23346	1:1500
anti-GPX4	Abmart	T56959	1:1000
anti-ACSL4	UpingBio	YP-Ab-12,589	1:1000
anti-xCT	MCE	HY-P80523	1:1000
anti-Ferritin heavy chain	MCE	HY-P80670	1:1000
anti-COX-2	Wanleibio	WL01750	1:1000
anti-HSPA5	Wanleibio	WL03157	1:1000
anti-NOX4	Wanleibio	WL06196	1:1000
anti-TLR4	Proteintech	66350-1-lg	1:3000
anti-occludin	Proteintech	27260-1-AP	1:10000
anti-claudin 1	Proteintech	13050-1-AP	1:3000
anti-ZO-1	UpingBio	YP-Ab-06280	1:1000
anti-NF-κB p65	Proteintech	80979-1-RR	1:10000
anti-phospho-NF-κB p65 (Ser536)	Abmart	TP56372	1:1000
anti-UCP1	Abclonal	A5857	1:1000
anti-PGC-1α	Abclonal	A12348	1:1000
anti-β-tubulin	Abmart	P60039S	1:6000
anti-β-actin	Boster	BM3873	1:12000

### 16 S rRNA sequencing

Total DNA was extracted from mouse feces. Nanodrop was used to quantify DNA, and 1.2% agarose gel electrophoresis was used to detect the quality of DNA extraction. Amplification of the V3-V4 region of microbial 16 S rRNA using paired primers (forward primer: 5′-ACTCCTACGGGAGGCAGCA-3′; reverse primer: 5′-GGACTACHVGGGTWTCTAAT-3′). After PCR amplification, purification and fluorescence quantification of target fragments, sequencing libraries were prepared using Illumina’s TruSeq Nano DNA LT Library Prep Kit. Frasergen completed the testing of the above experimental steps. Microbial diversity was assessed using alpha-diversity based on Chao1, Shannon and Simpson indices, microbial community changes were assessed using PCoA based on Bray-Curtis and Unweighted unifrac distances, and LDA Effect Size (LEfSe) was used to identify groups of mouse corresponding characteristic taxa.

### Cell experiment

#### Cell culture

The HepG2 cell line was purchased from the China Center for Type Culture Collection (Wuhan, China). The HepG2 cell line was cultured with high-glucose DMEM medium (Servicebio, China) that contained 10% fetal bovine serum (PAN, Germany) and 1% penicillin-streptomycin solution (New Cell & Molecular Biotech, China) at 37 °C with 5% CO_2_.

#### Reagent Preparation

As mentioned in previous literature, treating HepG2 cell lines with 200 µM palmitic acid (PA) for 24 h could construct a cell model of steatosis (Zhu et al. 2019). We prepared 200 µM PA (Sigma, USA) (containing 20% fatty-acid-free bovine serum albumin (BSA) (Biosharp, China). BSA was dissolved by gently vibrating with preheated PBS at 55 ℃. A stock solution was prepared by dissolving 20 mg lycorine in 1 mL DMSO (MCE, USA).

#### Cell counting Kit-8 (CCK-8) assay

When the density of HepG2 in the 96-well plate reached around 60%, cells were incubated with different concentrations of lycorine solution (0 µM + DMSO, 0.25 µM, 0.5 µM, 1 µM, 2 µM, 4 µM, 8 µM, 16 µM, 32 µM, 64 µM, 128 µM). After 24 h, aspirated the solution in the well and added 100 µL of diluted CCK-8 reagent (Sevenbio, China) to each well. After about 15 min, we detected the plate at a wavelength of 450 nm. Calculated the percentage of viable cells by comparing with control (untreated) cells.

#### Cell treatment

When the cells grew to 40-50% confluence, the steatosis hepatocyte model was established with the PA solution for 24 h, and the control group was treated with 20% fatty-acid-free BSA. At 24–48 h, mixed solutions of different concentrations of lycorine and 200 µM PA were added to HepG2 culture medium.

#### siRNA transfection

In 6-well plates, when the HepG2 fusion rate reached 40-50%, we transfected the cells with a siRNA targeting EGFR or with a scrambled siRNA (si-NC) (Wuhan GeneCreate Biological Engineering Co., Ltd, China), by using the gene transfection reagent, jetPRIME. After transfection for 24 h, BSA or 200 µM PA was added according to experimental requirements for 48 h. Then a certain concentration of lycorine was added between 48 and 72 h after transfection. The total duration of cell transfection and drug treatment was 72 h. The siRNA sequences were shown in Table 3.

**Table 3:** The sequence of small interfering RNA

Name	Forward sequence (5’→3’)	Reverse sequence (5’→3’)
si-NC	UUCUCCGAACGUGUCACGUTT	ACGUGACACGUUCGGAGAATT
si-EGFR	GGUCUUGAAGGCUGUCCAATT	UUGGACAGCCUUCAAGACCTT

si-NC: si-normal control; si-EGFR: si-epidermal growth factor receptor

#### Oil red O staining of HepG2

The samples were washed thrice with PBS and then fixed with 4% polyformaldehyde for 20 min. Washed twice with distilled water, then added 60% isopropanol for 15 min before discarding. After air-drying for 2 min, added the filtered oil red O working solution to avoid light staining for 20 min. After washing with 60% isopropanol for 5 s, rinsed thrice with distilled water. After staining the cell nucleus with hematoxylin for 90s, rinsed with distilled water until the liquid was clear. Sealing with glycerol gelatin, and then observed under a microscope.

#### qRT-PCR and Western blot

Added 400 µL FreeZol reagent to isolate total RNA or 120 µL RIPA lysis solution containing 1% PMSF and 1% phosphorylase inhibitor to prepare protein in each well of a 6-well plate. The remaining steps were the same as animal experiments.

### Statistical analysis

The data were analyzed by SPSS 20.0. All results were expressed as means ± SD. Student’s t-test was used for normally distributed data of two unpaired groups. For data between multiple groups, one-way ANOVA was used between data groups with normal distribution and homogeneous variance. Brown Forsythe and Welch ANOVA test was used if the variance was uneven. *P* < 0.05 was statistically significant.

## Results

### Lycorine reduced body weight and ameliorated hepatic steatosis in MASLD mice

After 12 weeks of normal diet and HFD feeding, the weight of the other four groups had significantly increased compared to the control group. At the end of the 16th week, we observed that all the MASLD mice weighed significantly less than those in the HFD group after 4 weeks of intervention with the 3 doses of lycorine (Fig. 1B). As shown in Fig. 1C, the livers of control mice were reddish, whereas the livers of HFD mice were significantly lighter in color than those of the control group, and the lesions were clearly visible to the naked eye. After lycorine intervention, the livers of mice were significantly redder in color than those of the HFD group, and no obvious lesions were visible to the naked eye. The results of HE and oil red O staining revealed that the HFD group showed obvious vacuoles and larger lipid droplets in the livers, and quantitative analysis indicated that the area of lipid droplets in the HFD group was significantly increased (Fig. 1D and E). After lycorine intervention, it was seen that hepatic steatosis was significantly reduced and lipid droplets became smaller and lipid droplet area decreased compared with the HFD group, and this improvement seemed to be enhanced with the increase in the dose of lycorine in this experiment, showing a dose-dependent effect.

**Fig. 2:**
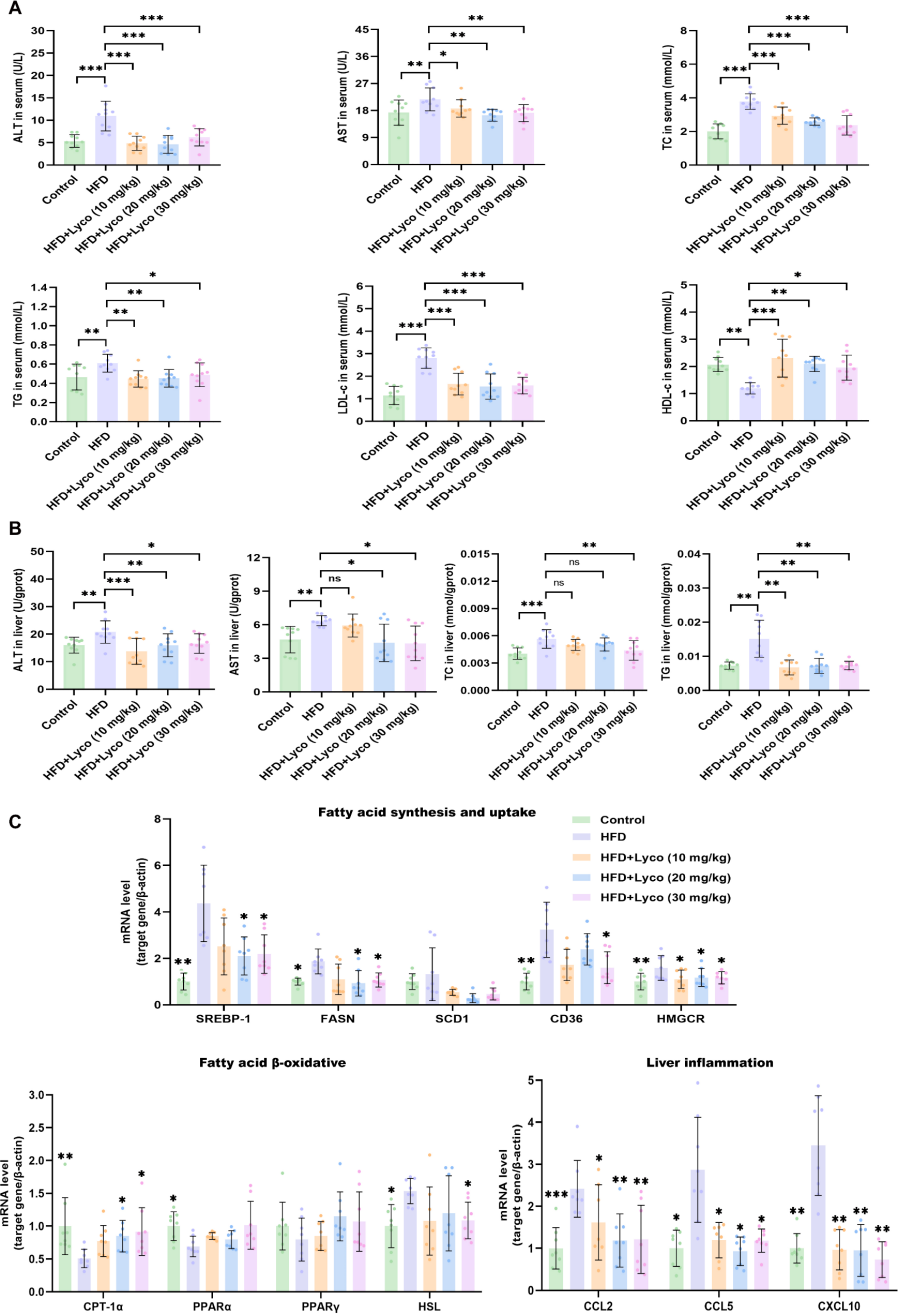
Lycorine attenuated hepatic steatosis in MASLD mice. (**A**) serum ALT, AST, TC, TG, LDL-c and HDL-c levels in mice (*n* = 10); (**B**) hepatic ALT, AST, TC, TG levels in mice (*n* = 10); (**C**) qRT-PCR results of genes related to fatty acid metabolism and inflammation in the liver of mice (*n* = 8). ^ns^*P* > 0.05, ^*^P < 0.05, ^**^P < 0.01, ^***^P < 0.001 vs. HFD

Serum liver function indexes showed that the levels of ALT, AST, TC, TG, and LDL-c were all significantly higher in the HFD group compared with the other four groups, while HDL-c was significantly lower (Fig. 2A). In addition, we examined the levels of ALT, AST, TC and TG in the liver. As shown in Fig. 2B, gavage of 30 mg/kg/d had the most significant effect as compared to low and medium doses of lycorine, which showed significant reduction in hepatic ALT, AST, TC and TG levels compared to the HFD group. The qRT-PCR results indicated that fatty acid synthesis and uptake related genes such as SREBP-1, FASN, CD36 and HMGCR were reduced by lycorine. Similarly, fatty acid β-oxidation related genes CPT-1α and HSL were significantly down-regulated by lycorine. Notably, although low and medium doses of lycorine did not downregulate all of the above indicators, high-dose lycorine significantly downregulated all of the above genes (Fig. 2C). Interestingly, neither dose of lycorine affected the mRNA expression levels of SCD1, PPARα, and PPARγ. In addition, the mRNA expression of inflammation-related genes CCL2, CCL5, and CXCL10 was significantly increased in the HFD group compared with the other four groups, and different doses of lyrcorine significantly attenuated hepatic inflammation in MASLD mice.

In conclusion, the above results confirmed that lycorine attenuated hepatic steatosis in MASLD with a dose-dependent effect on most indicators.

### Lycorine ameliorated hepatic OS and ferroptosis in MASLD mice

**Fig. 3:**
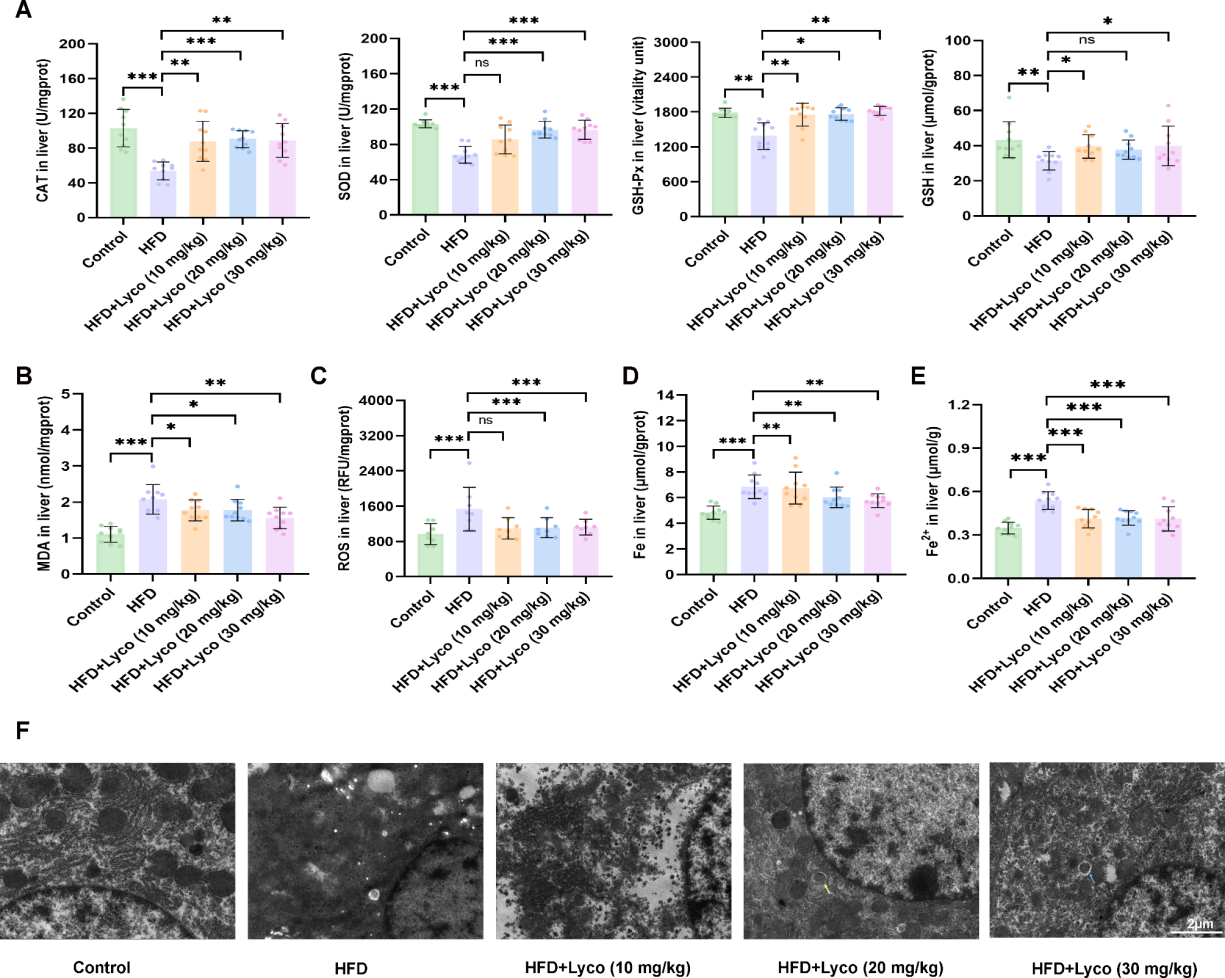
Lycorine attenuated hepatic oxidative stress and ferroptosis in MASLD mice. (**A**) hepatic antioxidant CAT, SOD, GSH-Px and GSH content (*n* = 10); (**B**) hepatic lipid peroxides MDA content (*n* = 10); (**C**) hepatic ROS content (*n* = 8); (**D**) hepatic iron content (*n* = 10); (**E**) hepatic ferrous iron content (*n* = 10); (**F**) hepatic TEM, yellow arrows are mitochondrial autophagosomes, blue arrows are autophagy-like structures (scale bar = 2 μm). ^ns^*P* > 0.05, ^*^P < 0.05, ^**^P < 0.01, ^***^P < 0.001 vs. HFD

OS is defined as an imbalance between ROS production and endogenous antioxidant defense systems (van der Pol et al. 2019). The present experiment indicated that the antioxidant enzymes CAT, SOD, GSH-Px activities and antioxidant GSH content were significantly increased and lipid peroxides MDA content was significantly decreased with high-dose lycorine compared with the HFD group. Although SOD and ROS levels in the low-dose group and GSH levels in the medium-dose group were not significantly different from those in the HFD group after four weeks of lycorine intervention, the rest of the indicators were significantly improved (Fig. 3A-C).

In addition, the liver iron and Fe^2+^ content in the HFD group was significantly higher than that of the remaining four groups, and lycorine significantly reduced the deposition of iron in the liver (Fig. 3D and E). TEM showed that the mitochondria in the HFD group had significantly more lipid droplets than those in the control group and the lycorine intervention group, and the morphology of mitochondria was significantly changed, with the outer mitochondrial membrane blurred and the mitochondrial cristae reduced or disappeared, but the nuclei had no obvious morphological changes, which was in line with the manifestation of ferroptosis. Individual mitochondrial degeneration occurred in the low-dose lycorine group, but most of the mitochondria had normal morphology. Interestingly, we observed mitochondrial autophagosomes (yellow arrows) in the medium-dose lycorine group and the mitochondrial morphology was significantly better than that of the low-dose group, and the mitochondrial morphology in the high-dose lycorine group was also significantly better than that of the HFD group and the low-dose group, and an autophagy-like structure (blue arrows) was observed (Fig. 3F). The above results suggested that lycorine could reduce hepatic iron deposition and attenuate hepatic iron death, and the improvement of MASLD appeared to be better in the middle-dose and high-dose lycorine than in the low-dose group.

### Network pharmacology, molecular docking, Western blot and ELISA results suggested that EGFR was the most likely potential target for lycorine to ameliorate MASLD

**Fig. 4:**
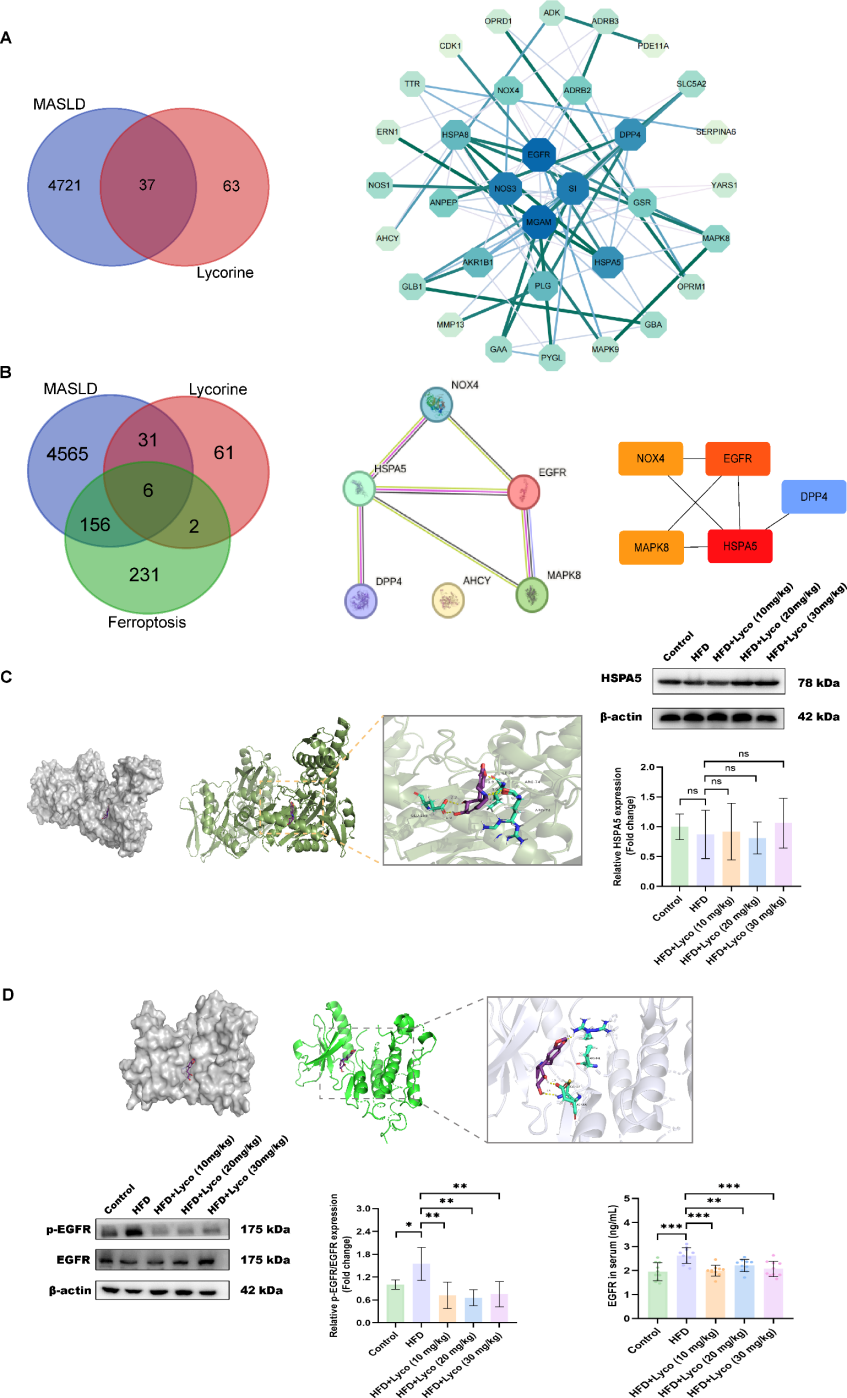
EGFR as a potential target for lycorine intervention in MASLD. (**A**) Venn diagram of genes intersected by MASLD and lycorine as well as PPI interaction network; (**B**) Venn diagram of genes intersected by MASLD, lycorine and ferroptosis as well as PPI interaction network; (**C**) docking site maps of ligand and receptor molecules (HSPA5) for lycorine treatment of MASLD and the liver protein expression levels of HSPA5 in each group of mice (*n* = 4); (**D**) docking site maps of ligand and receptor molecules (EGFR) for lycorine treatment of MASLD, the liver protein expression levels of p-EGFR/EGFR in each group of mice (*n* = 4) and serum EGFR expression levels in each group of mice (*n* = 10). The larger the node, the darker the color, indicating that the larger the Degree value, the greater the influence of the target on the MASLD. The thicker the connecting line between targets and the darker the color indicate the closer relationship between two targets. ^ns^*P* > 0.05, *P < 0.05, **P < 0.01, ***P < 0.001 vs. HFD

A total of 100 potential targets of lycorine were obtained from the SwissTarget Prediction database. A total of 4758 MASLD disease targets were screened in GeneCard, OMIM, DisGeNET, TTD and Drugbank. Through Venn diagram, we found 37 intersecting genes of lycorine and MASLD. The PPI results revealed that “EGFR” and “MGAM” had the largest nodes and the darkest colors with the same and largest Degree values, but “EGFR” had the largest Betweenness, and Closeness values than “MGAM” (Fig. 4A). Degree values were the same and the largest, but the Betweenness, and Closeness values of “EGFR” were larger than those of “MGAM”. Therefore, we believed that the binding ability of lycorine to EGFR is better than that of lycorine to MGAM.

We also plotted the ferroptosis-related targets downloaded from the FerrDb with the MASLD and lycorine targets. As shown in Fig. 4B, there are six intersecting genes, which were imported into the STRING database platform and found that “AHCY” was not associated with the other five genes. The PPI network of the five related genes was plotted and scored by Cytoscape, and the redder the color means the higher the score. The results showed that “HSPA5” and “EGFR” had the highest scores.

In order to verify the strong binding ability of lycorine to HSPA5 and EGFR, we obtained the X-ray crystal structures of HSPA5 (PDB ID: 5EX5) and EGFR (PDB ID: 8A27) from the RCSB PDB database. The molecular docking results indicated that the binding energy size of lycorine with HSPA5 was − 5.52 kcal/mol, and that with EGFR was − 5.25 kcal/mol. Moreover, lycorine bound to both HSPA5 and EGFR with hydrogen bonding interactions. It is generally believed that the binding of ligand and receptor <-5 kcal/mol proves the better binding activity. This showed that lycorine had a good ability to bind to both HSPA5 and EGFR. Visualization indicated that lycorine bound to the active site of HSPA5 protein and formed hydrogen-bonding interactions with ARG-74 (lengths: 3.0 Å and 3.1 Å), ILE-76 (length: 1.9 Å), and GLU-155 (lengths: 1.9 Å and 2.2 Å)(Fig. 4C). Lycorine also bound to the EGFR protein active site and formed hydrogen bonding interactions with ARG-841 (length: 1.8 Å), CYS-797 (length: 2.5 Å) and ASP-800 (length: 1.9 Å)(Fig. 4D). Therefore, we concluded that both HSPA5 and EGFR have a strong binding capacity to lycorine.

To evaluate which of the two proteins is more likely to be a potential target for lycorine therapy for MASLD, we performed western blot, and the results confirmed that the protein expression of HSPA5 was not significantly different between the groups (Fig. 4C). While p-EGFR/EGFR was significantly elevated in the HFD group, the ratios of p-EGFR/EGFR were significantly decreased after the intervention of all three doses of lycorine compared with that of the HFD group (Fig. 4D). Besides, the serum EGFR levels of mice also showed the same results as western blot, with the highest expression level of EGFR in the HFD group (Fig. 4D).

A study showed that EGFR mutant cells were sensitive to ferroptosis (Poursaitidis et al. 2017). Other studies also indicated that EGFR could promote ferroptosis in breast cancer (Wu et al. 2022) and glioblastoma (Wu et al. 2023). In addition, EGFR was labeled as a driver of ferroptosis on the FerrDb website, and our experiment also confirmed that EGFR expression was significantly elevated in the HFD group. In conclusion, we believed that EGFR was the most likely potential target for lycorine to improve MASLD.

### Lycorine attenuated hepatic OS and ferroptosis in MASLD mice by affecting the EGFR/PI3K/AKT signaling pathway

**Fig. 5:**
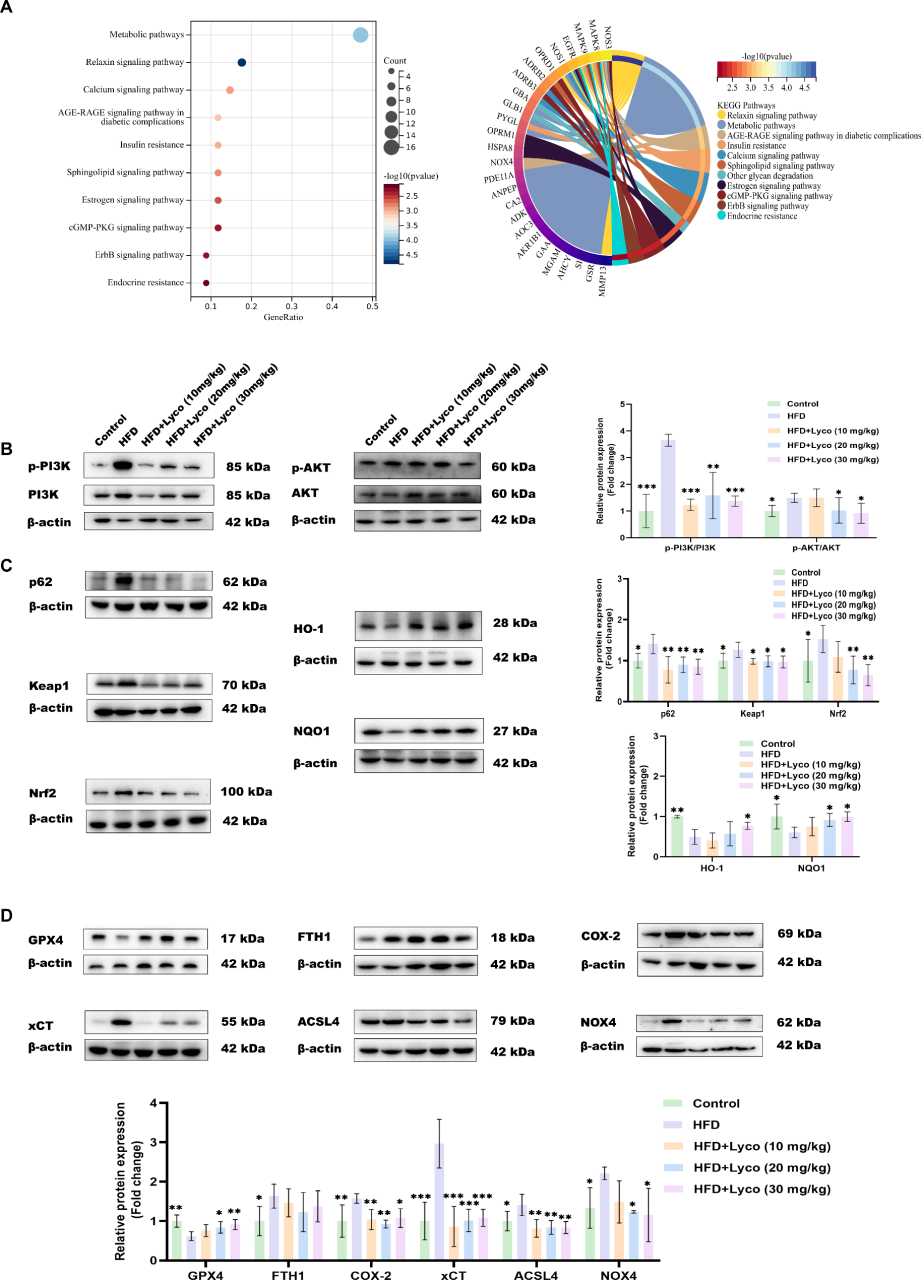
Lycorine ameliorated hepatic oxidative stress and ferroptosis in MASLD mice through the EGFR/PI3K/AKT pathway. (**A**) bubble and circle plots of KEGG signaling pathway of MASLD with lycorine cross-talk genes; (**B**) protein expression levels of p-PI3K/PI3K, p-AKT/AKT in the livers of various groups of mice (*n* = 3–5); (**C**) expression of OS-related proteins in liver (*n* = 4–5); (**D**) expression of ferroptosis-related proteins in liver (*n* = 3–4). ^ns^*P* > 0.05, ^*^P < 0.05, ^**^P < 0.01, ^***^P < 0.001 vs. HFD

The 37 intersecting targets of MASLD and lycorine were imported into the online analysis software Sangerbox for KEGG pathway analysis. As in Fig. 5A, all ten KEGG pathways in the bubble diagram displayed significance, and the circled diagram showed that EGFR was associated with five of the KEGG pathways. In addition, by reviewing the KEGG website we found that all of the above ten pathways were associated with either EGFR or PI3K/AKT. Subsequently, we examined the protein ratios of hepatic p-PI3K/PI3K and p-AKT/AKT. The results confirmed that the protein ratios of hepatic p-PI3K/PI3K and p-AKT/AKT in MASLD mice were both significantly lower than those in the HFD group after the intervention of intermediate and high doses of lycorine (Fig. 5B). Interestingly, although the protein ratio of p-PI3K/PI3K was significantly lower in the low-dose lycorine group compared with the HFD group, the ratio of p-AKT/AKT was not significantly different between the two groups. Therefore, we hypothesized that lycorine may intervene in MASLD by targeting EGFR and thus affecting the PI3K/AKT signaling pathway, and that this ameliorative effect on MASLD was dose-regulated by lycorine.

Our study also found that lycorine reduced the protein expression of liver p62, keap1 to nrf2, and increased the expression of NQO1 and HO-1, anti-OS-related proteins, in MASLD mice (Fig. 5C). In addition, we examined ferroptosis-related proteins, and although FTH1 protein expression was not significantly different between the HFD group and the lycorine intervention group after lycorine intervention, lycorine increased the expression of GPX4, a marker protein related to ferroptosis, and decreased the protein expression of COX-2, xCT, ACSL4, and NOX4 (Fig. 5D). Combining all the results of Fig. 5B-D, we concluded that there may be a dose-dependent effect of lycorine on the amelioration of hepatic OS and ferroptosis in MASLD mice, and that the amelioration of MASLD appeared to be stronger at higher doses of lycorine in the present experiment.

### Lycorine exerted anti-MASLD effects via EGFR

**Fig. 6:**
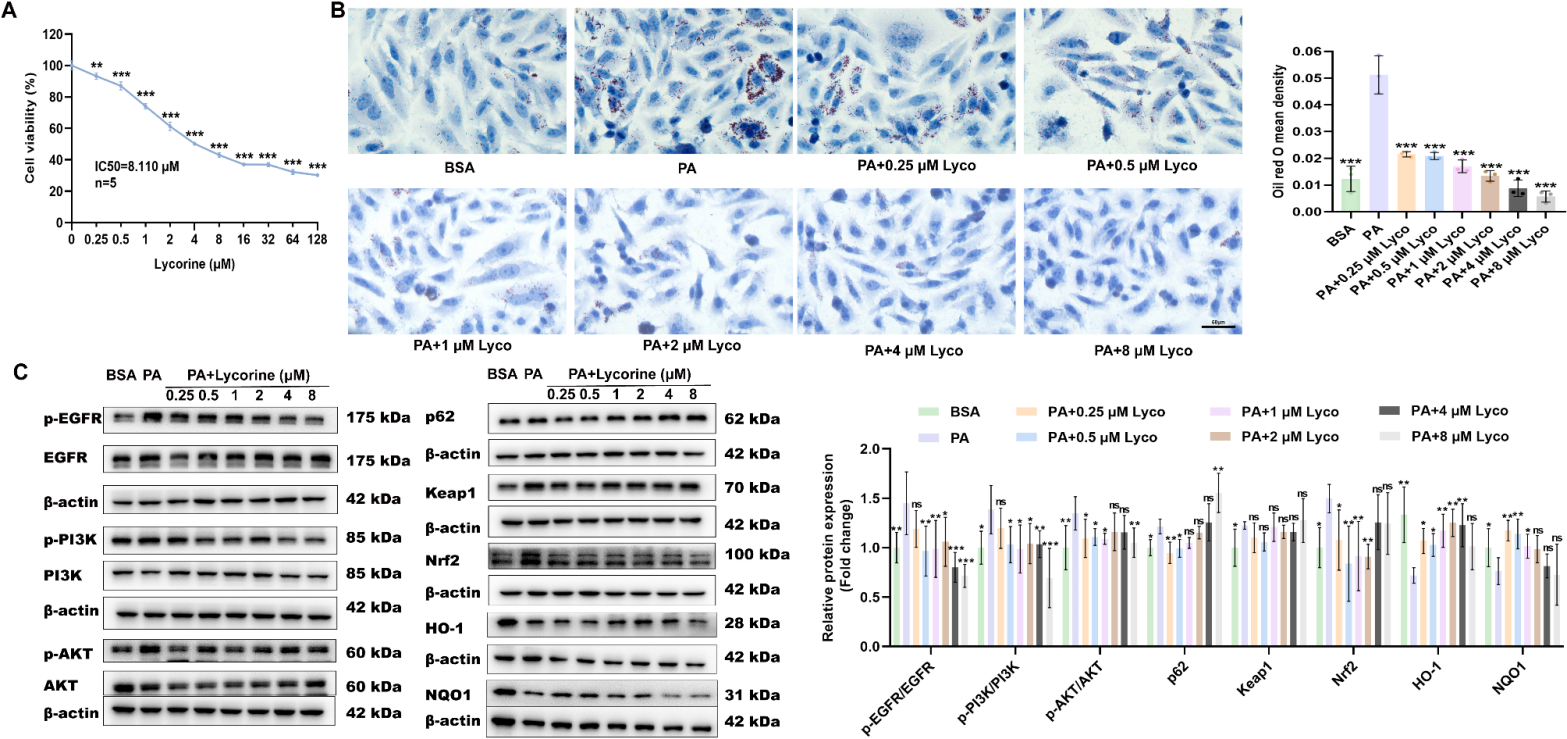
The effect of different concentrations of lycorine on HepG2 vitality and its mechanism of impact on HepG2 steatosis. (**A**) cell counting kit-8 (*n* = 5); (**B**) oil red O staining of HepG2 (*n* = 3); (**C**) protein expression levels of EGFR/PI3K/AKT pathway and its downstream (*n* = 3–5). ^ns^*P* > 0.05, ^*^P < 0.05, ^**^P < 0.01, ^***^P < 0.001 vs. PA

We used CCK-8 to detect the cell viability of HepG2 cells treated with different concentrations of lycorine (0, 0.25, 0.5, 1, 2, 4, 8, 16, 32, 64, 128 µM) for 24 h. The results showed that lycorine reduced the activity of HepG2 in a dose-dependent manner, and the IC50 value was 8.11µM (Fig. 6A). According to the results of cell viability, the subsequent experiments adopted a treatment regimen of 0, 0.25, 0.5, 1, 2, 4, and 8 µM for HepG2 cells for 24 h.

The results of oil red O indicated that 200 µM PA-treated HepG2 steatosis was obvious. After 24 h of treatment with different concentrations of lycorine, the lipid droplets gradually decreased with the increase of lycorine concentration (Fig. 6B). The results of western blot were also similar to animal experiments, although there was no significant difference in p-EGFR/EGFR and p-PI3K/PI3K between the PA group and PA + 0.25 µM lycorine group, significant differences were observed after 0.5-8 µM lycorine intervention. Based on the protein expression results of p-AKT/AKT in Fig. 6C, we believed that lycorine could improve MASLD through the EGFR/PI3K/AKT signaling pathway. It was worth noting that although the protein expression of HO-1 increased with the increase of lycorine concentration, the protein expression of p62, keap1, and nrf2 showed a trend of first decreasing and then increasing, while the protein expression of NQO1 showed a trend of first increasing and then decreasing (Fig. 6C). Above results indicated that although low concentrations of lycorine could improve steatosis in HepG2, when the concentration exceeds a certain range, the protective effect of lycorine on steatosis in HepG2 seemed to decrease, and even aggravated the damage. Based on the effects of different concentrations of lycorine on HepG2 cell viability, on the EGFR/PI3K/AKT signaling pathway and its downstream proteins in steatotic hepatocytes, we concluded that 0.5 µM was the optimal concentration of lycorine to intervene in steatotic HepG2 in this experiment.

**Fig. 7:**
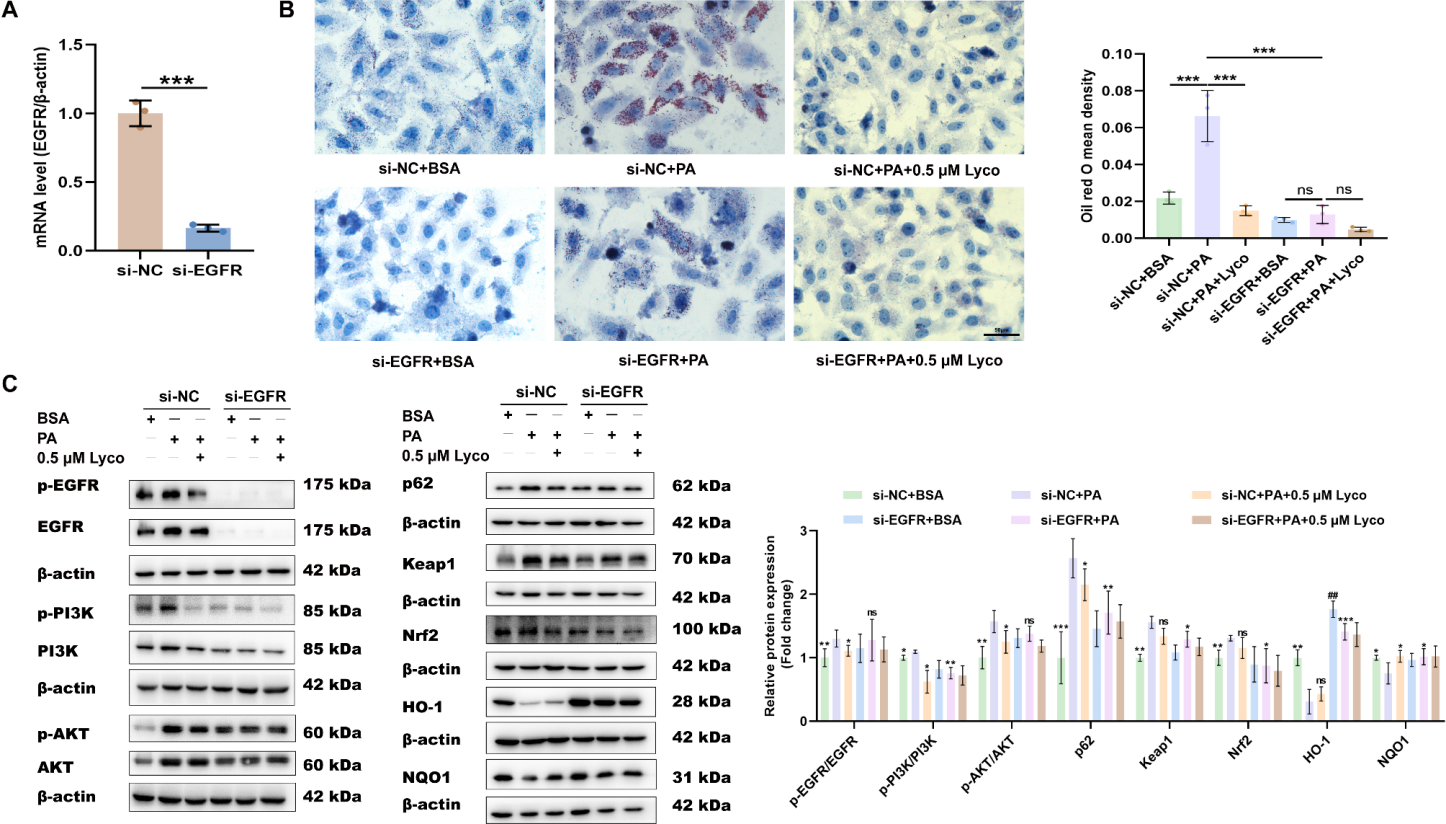
The role of EGFR in treating hepatic steatosis with lycorine. (**A**) mRNA level of EGFR after siRNA transfection 24 h (*n* = 3); (**B**) oil red O staining (*n* = 3); (**C**) protein expression levels of EGFR/PI3K/AKT pathway and its downstream (*n* = 3–5). ^ns^*P* > 0.05, ^*^P < 0.05, ^**^P < 0.01, ^***^P < 0.001 vs. si-NC + PA; ^##^*P* < 0.01 vs. si-EGFR + PA

To further clarify lycorine’s impact on EGFR and downstream signaling, we used si-EGFR. After 24 h of transfection, we detected the level of EGFR by qRT-PCR and found that the EGFR level was significantly decreased after being treated with si-EGFR in HepG2 (Fig. 7A), indicating successful transfection. The oil red O results revealed that knocking down EGFR significantly reduced the lipid droplets induced by PA, and there was no significant difference in lipid droplets when given BSA, PA, or PA + 0.5 µM lycorine based on knocking down EGFR (Fig. 7B). The results of western blot also suggested that the EGFR/PI3K/AKT signaling pathway was inhibited, its downstream proteins aggravating MASLD injury were decreased, and the expression of anti-oxidative stress-related proteins was increased in the si-EGFR + PA group compared with the si-NC + PA group (Fig. 7C). In contrast, there was no significant difference in the expression of the EGFR/PI3K/AKT signaling pathway and its downstream proteins in the si-EGFR + PA group compared with the si-EGFR + PA + 0.5 µM group (Fig. 7C). Taken together, these results indicated that EGFR was the target for lycorine intervention in MASLD.

### Lycorine promoted WAT and BAT energy metabolism

**Fig. 8:**
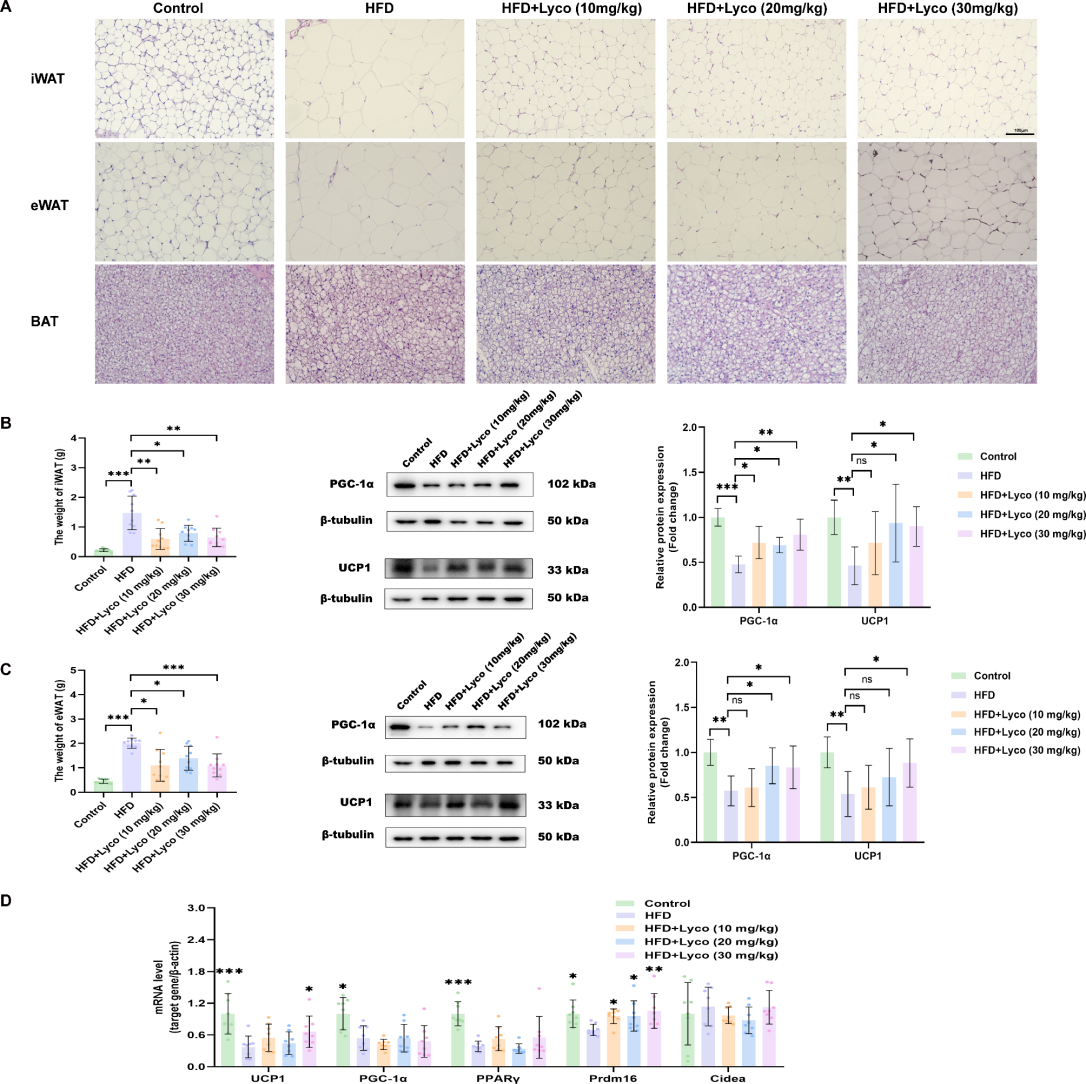
Lycorine enhanced adipose tissue energy metabolism. (**A**) HE staining of iWAT, eWAT and BAT; (**B**) iWAT weight (*n* = 10) and PGC-1α and UCP1 protein expression levels (*n* = 4–5); (**C**) eWAT weight (*n* = 10) and PGC-1α and UCP1 protein expression levels (*n* = 4–5); (**D**) mRNA expression levels of thermogenesis-related genes in BAT (*n* = 8). ^ns^*P* > 0.05, ^*^P < 0.05, ^**^P < 0.01, ^***^P < 0.001 vs. HFD

After opening the abdominal cavity of the mice, a large amount of WAT was visible in the HFD group, while WAT was significantly reduced in the other four groups (Fig. 1C). HE staining results also confirmed that all three doses of lycorine reduced the cell size of iWAT and eWAT in MASLD mice (Fig. 8A). After weighing, we also found that lycorine decreased the weight of WAT (Fig. 8B and C). Compared with mice in the HFD group, lycorine increased the expression levels of UCP1 and PGC-1α proteins in iWAT and eWAT, and the effect was particularly significant at high-dose lycorine (Fig. 8B and C).

BAT consumes energy through thermogenesis, and we observed that the adipocytes of BAT in MASLD mice after lycorine intervention became smaller and contained abundant multi-atrial lipid droplets (Fig. 8A). The qRT-PCR results indicated that all three doses of lycorine increased the level of Prdm16 in the BAT of MASLD mice, although the thermogenic gene Cidea, the lipogenic gene PPARγ, and the mitochondrial biogenesis gene PGC-1α did not show significant changes in the BAT after lycorine intervention compared with the HFD group. Notably, the high-dose lycorine also induced an increase in the level of UCP1 in the BAT of MASLD mice (Fig. 8D). The protein levels of UCP1 and Prdm16 in BAT reflect thermogenic capacity, and UCP1 must be activated to increase thermogenesis (Wang and Seale 2016; Harms et al. 2014). Therefore, we hypothesized that high-dose lycorine could stimulate the thermogenic function of BAT to convert energy into heat.

### Lycorine modulated the composition of intestinal flora in MASLD mice

**Fig. 9:**
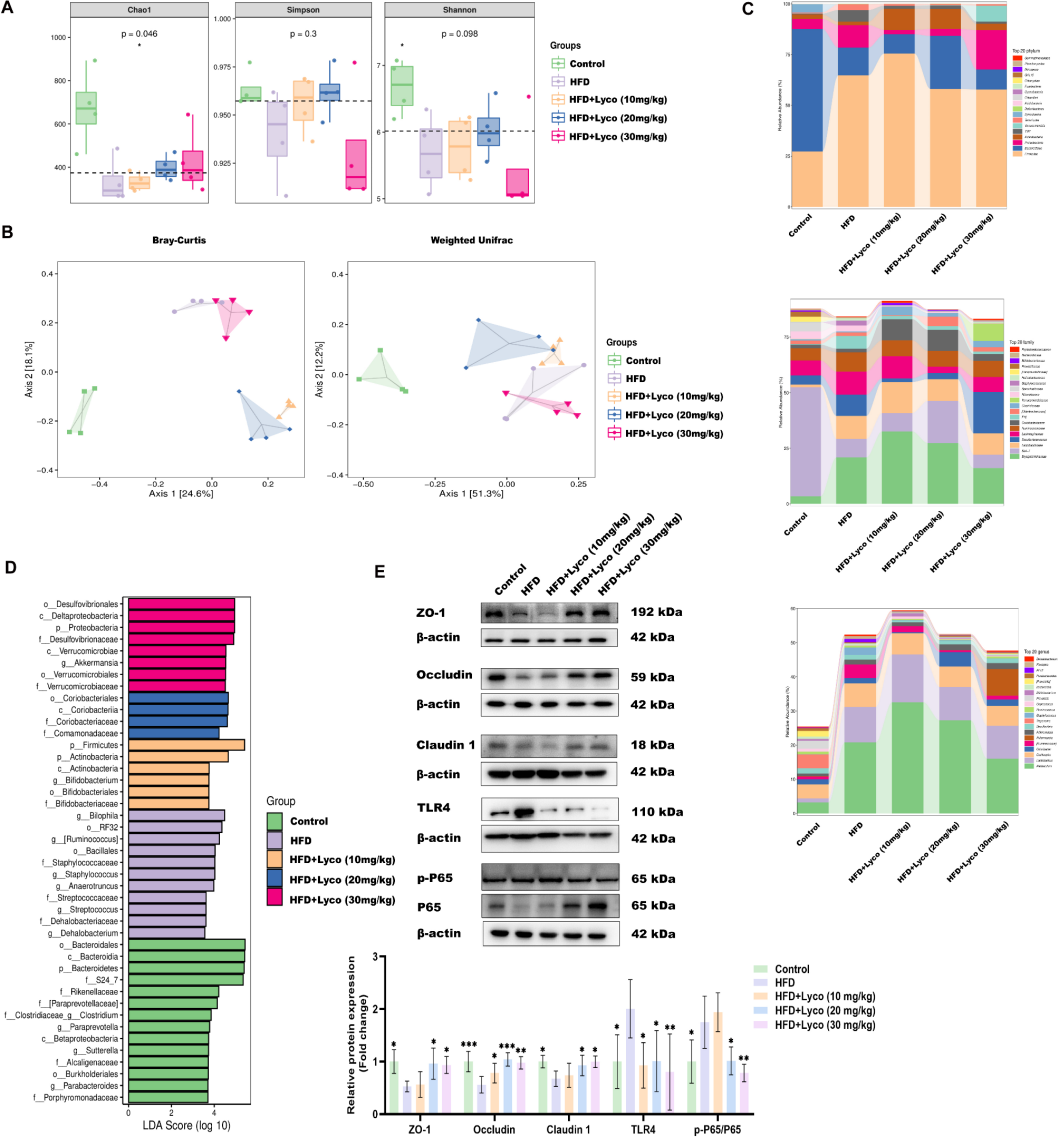
Lycorine may affect the intestinal tract of MASLD mice. (**A**) α-diversity indexes Chao1, Shannon, and Simpson (*n* = 4); (**B**) PCoA based on Bray-Curtis and Weighted unifrac (*n* = 4); (**C**) changes in gut microbiota between different groups at phylum, family and genus levels; (**D**) LEfSe analysis based on LDA score of significantly different species (*n* = 4); (**E**) colonic ZO-1, Occludin, Claudin 1, TLR4 and p-P65/P65 protein expression level (*n* = 3–5). *P < 0.05, **P < 0.01, ***P < 0.001 vs. HFD

To investigate the effects of HFD and lycorine treatment on the gut microbiota, we analyzed mouse feces by 16 S rRNA sequencing. α-diversity index Chao1 and Shannon’s results showed that a HFD decreased the total number of species and abundance of intestinal flora. Simpson revealed that species homogeneity in the HFD group and the high-dose lycorine group was lower than that in the the other three groups (Fig. 9A). In addition, β-diversity (PCoA of Bray-Curtis and weighted unifrac) annotated the differences in community composition (Fig. 9B). We found that the HFD significantly affected the intestinal flora species in mice and that the difference in the intestinal flora of MASLD mice compared to the HFD group was significant after low and medium-dose lycorine interventions. Interestingly, the composition of the intestinal flora in the high-dose lycorine group was closer to that of the HFD group, echoing the results of the species composition analysis.

We quantified the relative abundance of microbial taxa at multiple levels to identify characteristic microorganisms for each group. At the phylum level, all four groups consuming a HFD exhibited an increased ratio of *Firmicutes*/*Bacteroidetes* compared with the control group. The lycorine intervention increased the abundance of *Actinobacteria* and decreased the abundance of *TM7* compared to the HFD group. Notably, *Verrucomicrobia* abundance was significantly increased in the high-dose lycorine group compared to the other four groups. At the family level, *F16* abundance was significantly higher in the HFD group. The abundance of *Desulfovibrionaceae*, the opportunistic pathogen, was reduced after low and medium-dose lycorine interventions compared to the HFD and high-dose lycorine groups, whereas the abundance of *Coriobacteriaceae* was increased. Interestingly, as with the phylum level, we again saw a concomitant increase in the abundance of both opportunistic pathogens and beneficial intestinal bacteria in the high-dose lycorine group (*Desulfovibrionaceae* and *Verrucomicrobiaceae* abundance were both increased compared to the other four groups). At the genus level, we observed a significant increase in *Akkermansia* in the high-dose lycorine group (Fig. 9C).

In addition, LEfSe analysis showed that *Bacteroidetes* were the biomarkers with the most significant difference between the control group and the other groups. Fat-loving, bile-resistant, pro-inflammatory *Bilophila* was the biomarker in the HFD group. After after lycorine treatment, *Firmicutes* and *Actinobacteria* were the two phylum that differed significantly in the low-dose lycorine group compared to the other groups, with *Bifidobacterium* being the biomarkers. In the medium-dose lycorine group, *Actinobacteria* of the *Coriobacteriaceae* and *Comamonadaceae* showed significant differences compared to other groups. In the high-dose lycorine group, *Dehalobacterium* in *Proteobacteria* and *Akkermansia* in *Verrucomicrobia* exhibited significant differences from the other groups (Fig. 9D). Our results indicated significant changes in the species composition of the gut microbiota of HFD mice and an increase in the production of some of the beneficial intestinal bacteria after lycorine intervention, suggesting that the gut microbiota may be involved in mediating the effects of lycorine treatment.

### Lycorine enhanced colonic barrier protein expression and reduced intestinal inflammation

By western blot results, we found that the expression of intestinal barrier proteins ZO-1, occludin, and claudin 1 was significantly increased in both the medium-dose and high-dose lycorine groups compared with the HFD group (Fig. 9E). In addition, we examined intestinal inflammation-related proteins, and the results showed that all three doses of lycorine reduced the expression of TLR4 in MASLD mice, and the ratio of NF-κB p-P65/P65 was significantly reduced after the intervention of medium and high doses of lycorine compared with that in the HFD group. Notably, low-dose lycorine seemed to have no significant effect on the ratio of NF-κB p-P65/P65. The above results suggested that the effects of lycorine in enhancing colonic barrier protein expression and reducing intestinal inflammation were enhanced with increasing drug concentration in this experiment.

## Discussion

Our study demonstrated that in vitro, knockdown of EGFR attenuated PA-induced hepatocellular steatosis, and on this basis, the therapeutic role of lycorine in steatosis was limited. In vivo, lycorine has an interventional effect on multiple tissues in MASLD. It may impact hepatic steatosis, OS and ferroptosis in MASLD by mediating EGFR and consequently affecting the PI3K/AKT signaling pathway. Lycorine also promoted WAT browning to promote thermogenesis to consume fatty acids, and reduced WAT and BAT cell size. In addition, lycorine regulated intestinal flora, improved the intestinal barrier, and reduced intestinal inflammation. It is worth noting that the ameliorative effect of lycorine on MASLD had dose-dependent effects in many indicators. In this experiment, it seemed that the ameliorative effect of lycorine on MASLD was better as the drug dose increased. In conclusion, we have identified lycorine as a promising candidate for the treatment of MASLD, and EGFR was the target for lycorine intervention in MASLD.

Lycorine has a typical alkaloidal tetracyclic skeleton with a simple chemical structure. Therefore, it is easy to analyze the structure-function relationship based on its chemical structure. Through network pharmacology, molecular docking, and western blot validation in vivo and vitro, we identified EGFR as a potential therapeutic target for lycorine in the treatment of MASLD. EGFR, also known as ErbB-1, is a classical receptor tyrosine kinase belonging to the ErbB receptor family and one of the highly expressed proteins in the liver (Bhushan and Michalopoulos 2020). Several studies have now shown that EGFR is a potential target for intervention in MASLD, but its role in MASLD is still controversial. It was indicated that hepatic EGFR expression was significantly reduced in MASLD mice (Shao et al. 2023). However, a study found the total and phosphorylated expression levels of EGFR were higher in the livers of NASH patients (Song et al. 2021). Animal experiments also confirmed that EGFR was phosphorylated in the liver of MASLD mice, and inhibition of EGFR suppressed diet-induced hepatic lipid accumulation, OS, hepatic fibrosis and improved glucose tolerance in mice (Bhushan et al. 2019; Liang et al. 2018). This is similar to our results that HFD induced activation of hepatic EGFR phosphorylation and exacerbated MASLD in mice. Studies showed that there was a close interaction between EGFR and the PI3K/AKT signaling pathway (Zaryouh et al. 2022; Tiemin et al. 2020). Meanwhile, our results also showed that PI3K/AKT was significantly enriched in the signaling pathway with a high frequency of occurrence of the pathway. In vitro, combined with the results of western blot that after knocking down EGFR, the expression of PI3K and AKT also decreased accordingly, we speculated that lycorine could target EGFR and affect the PI3K/AKT signaling pathway to ameliorate MASLD.

Nrf2 is a key regulator of OS and inflammation, and its protein products help to control important biological processes related to the reduction of ROS and defense against OS (Li et al. 2018). Keap1 is a negative regulator of nrf2. Under normal conditions, newly synthesized nrf2 proteins are short-lived, are sequestered in the cytoplasm by keap1, and are rapidly ubiquitinated and degraded. A small fraction of nrf2 escapes degradation and is transported to the nucleus to act on the ARE, which activates the expression of antioxidant genes (e.g., NQO1, HO-1, GSS, and GCL) to resist external harmful stimuli and reduce ROS production (Tebay et al. 2015; Shen et al. 2020; Gao et al. 2021). p62 competes with nrf2 for binding to keap1, leading to dissociation of nrf2 from keap1. p62 expression was elevated in MASLD, keap1 was sequestered by p62 and no longer binds to nrf2, and the inhibitory effect of keap1 on nrf2 was reduced leading to increased nrf2 signaling. Due to overstimulation or chronic activation, nrf2 may convert its cytoprotective effects into cytotoxicity, leading to an imbalance in the antioxidant system (e.g., decreased levels of the antioxidant enzymes SOD, CAT, and GSH-Px, and an increase in MDA) and ultimately exacerbating MASLD.

GPX4 plays a major role in blocking ferroptosis by eliminating phospholipid hydroperoxides (Xue et al. 2023). In MASLD, when cellular cystine transport proteins are inhibited, intracellular GSH is depleted, which ultimately leads to the inactivation of GPX4 and the accumulation of lipid peroxidation, which reaches a certain level and induces cell death (Yang and Stockwell 2016). One of the features of ferroptosis is the excessive accumulation of lipid peroxides (Pope and Dixon 2023). MDA and NOX4 can induce lipid peroxidation via OS, which in turn exacerbates ferroptosis (Park et al. 2021). Futhermore, Stockwell found that several genes, such as COX-2, xCT, ACSL4, were induced during ferroptosis (Stockwell 2022). Combined with our experimental results, it can be concluded that lycorine ameliorates MASLD ferroptosis.

MASLD is caused by dysfunction of adipose tissue and an imbalance between metabolic disorders and inflammatory stress induced by insulin resistance and the defense and repair mechanisms of the steatotic liver (Zeng et al. 2024). The fat is classified as brown, white and beige. Brown adipocytes in BAT are characterized by the presence of multilocular lipid droplets and dense mitochondria containing UCP1, a key protein regulating thermogenesis in BAT. Mice with elevated brown fat thermogenesis are protected from many deleterious metabolic effects triggered by a HFD, including obesity and insulin resistance (Stanford et al. 2013; Kopecky et al. 1995). WAT is a major organ for storing and releasing energy. Beige adipocytes are present in various WAT reservoirs, and their development is more vigorous in iWAT than eWAT, with higher levels of brown adipokine expression (Wang and Seale 2016). Beige adipocytes have a BAT-like thermogenic function by activating the brown fat-specific protein UCP1, which promotes energy metabolism and reduces the amount of fatty acids entering the liver, and thus attenuates MASLD.This may be the reason why the UCP1 protein expression level of the medium-dose lycorine group was significantly increased in iWAT compared with that of the HFD group, but was not significantly different from that of the HFD group in eWAT.

Dysregulation of the intestinal barrier and microbiota may lead to metabolic disorders and autoimmune-related diseases (Martel et al. 2022). The expression of intestinal barrier proteins was restored after lycorine intervention in this experiment, suggesting that lycorine can improve the intestinal barrier. A systematic review reported that the ratio of Firmicutes/Bacteroidetes in were higher (Crovesy et al. 2020), which is consistent with the results at the gate level in this experiment, i.e., this ratio was reduced in the four groups that consumed a HFD. Increased abundance of *Actinobacteria* and *Lactobacillaceae* in the intestine has been reported to be associated with antisteatotic and antiinflammatory (Saeb et al. 2022). Several studies have also confirmed the benefits of *Akkermansia* in improving liver fat accumulation and inflammation in MASLD (Han et al. 2022, 2023). Whereas *F16* and *Desulfovibrionaceae* are closely associated with liver inflammation (Chen et al. 2020), both flora were increased in the HFD group. In addition, we examined the expression of the intestinal inflammation-related proteins TLR4 and NF-κB p-P65 and p65. TLR4 is an important member of the pattern recognition receptor family and is a key sensor of changes in the intestinal microbiota. the TLR4 /NF-κB p65 pathway is often used to assess changes in intestinal inflammation (Li et al. 2020). Our study revealed that this pathway was inhibited after lycorine intervention, indicating a reduction in the level of intestinal inflammation. Combined with changes in fecal intestinal flora and protein expression levels, we found an increase in the proportion of some beneficial intestinal bacteria, enhanced expression of intestinal barrier proteins and reduced expression of inflammatory proteins after lycorine intervention. Therefore, we hypothesized that lycorine could regulate intestinal homeostatic imbalance.

This experiment also has some limitations. First, only three drug concentrations were set up for gradient test, which could not determine the optimal dose range of lycorine to improve MASLD. Second, only animal experiments were conducted in this study, and in the future, we will collect serum or liver tissues from MASLD patients and healthy individuals for EGFR expression detection. Third, there are still some difficulties in obtaining genus and species identification through 16 S rRNA gene sequencing. In further study, we will attempt to use methods such as whole genome sequencing to more accurately explain the effects of lycorine on gut microbiota composition, and how gut microbiota modulation is connected to physiological changes in the liver and WAT at the species level.

## Conclusions

**Fig. 10:**
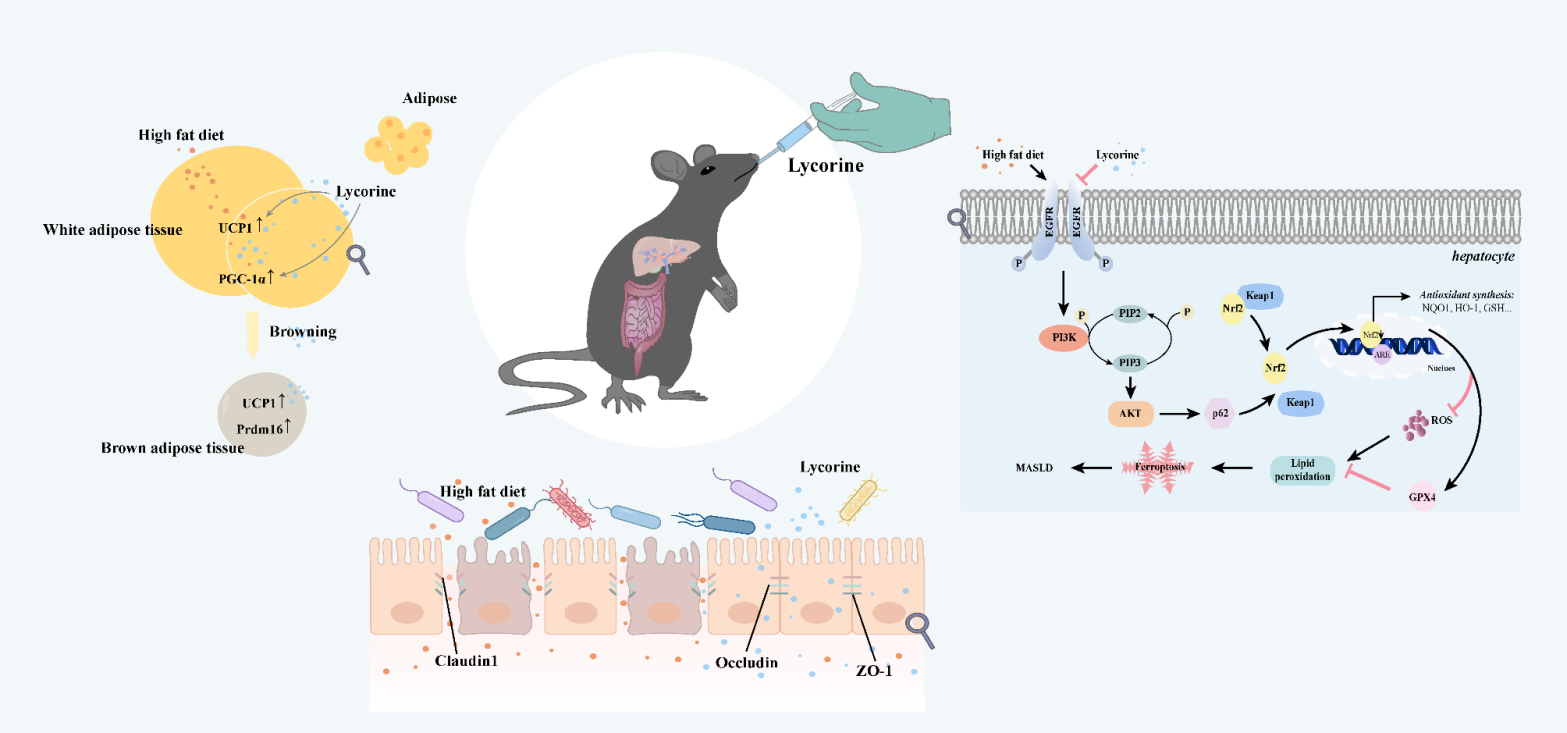
Mechanism diagram of intervention of lycorine on liver, intestine, and adipose tissue of MASLD mice. Lycorine inhibited high-fat diet-induced p-EGFR activation, which in turn inhibited PI3K/AKT signaling pathway and the expression of p62, keap1 and nrf2 in the cytoplasm. Increased nrf2 entering the nucleus enhanced antioxidant synthesis and inhibited ROS production to reduce oxidative stress. In addition, lycorine promoted the expression of GPX4, which, combined with attenuated ROS, inhibited lipid peroxidation and ameliorated hepatic ferroptosis in MASLD mice. For the intestine, lycorine enhanced colonic barrier protein expression (ZO-1, claudin 1 and occludin) and regulated the composition of intestinal microbiota. In addition, lycorine promoted WAT browning and thus thermogenesis for energy consumption

Our study demonstrated that EGFR was the target of lycorine intervention in MASLD. Lycorine could ameliorate hepatic steatosis, OS and ferroptosis in MASLD mice by inhibiting the expression of phosphorylated EGFR, which in turn inhibited the PI3K/AKT signaling pathway. In addition, lycorine promoted WAT browning and thus thermogenesis for energy consumption, and affected the composition of intestinal flora, improved the intestinal barrier, and reduced intestinal inflammation (Fig. 10). And the above effects may have a dose-dependent effect. Our study provides a new possibility for the treatment of MASLD.

## Electronic supplementary material

Below is the link to the electronic supplementary material.

Supplementary Material 1

## Data Availability

No datasets were generated or analysed during the current study.

## Abbreviations

BSA: Bovine serum albumin
CCK-8: Cell counting kit-8
MASLD: Metabolic dysfunction-associated steatotic liver disease
OS: Oxidative stress
ROS: Reactive oxygen species
GPX4: Glutathione peroxidase 4
HFD: High-fat diet
HE: Hematoxylin and eosin
eWAT: Epididymal white adipose tissue
iWAT: Inguinal white adipose tissue
BAT: Brown adipose tissue
TEM: Transmission electron microscopy
CAT: Catalase
GSH: Glutathione
GSH-Px: Glutathione peroxidase
MDA: Malondialdehyde
SOD: Superoxide dismutase
siRNA: Small interfering RNA
PA: Palmitic acid
PPI: Protein-protein interaction
EGFR: Epidermal growth factor receptor
FTH1: Ferritin heavy chain-1
