# Supplementary material for: Lycorine ameliorates liver steatosis, oxidative stress, ferroptosis and intestinal homeostasis imbalance in MASLD mice
Source: Mol Med. 2024 Nov 27;30:235. doi: 10.1186/s10020-024-01003-6 (PMC11600876; doi:10.1186/s10020-024-01003-6)
Supplement: Supplementary file 1 — Supplementary Material 1 [file 10020_2024_1003_MOESM1_ESM.docx]

**Measurement of catalase (CAT), glutathione peroxidase (GSH-Px), glutathione (GSH), superoxide dismutase (SOD) activities and malondialdehyde (MDA) levels**

Approximately 50 mg of liver tissue was weighed, and 10% liver homogenate was prepared by centrifugation at 3000 r/min and 4°C for 10 min. Then 0.5% liver homogenate was prepared by dilution with physiological saline to measure CAT and GSH-Px activities. In sequence, the reagents were added and mixed well, and 200 μL was taken and read at 405 nm on the enzyme marker.

A 10% liver tissue homogenate was prepared to measure the level of GSH. Then, 100 μL reagent 1 was added, and the homogenate was centrifuged at 24°C, 3500 r/min for 10 min. Took 100ul of supernatant and added reagents three and two in sequence to a 96-well plate. Let it stand at room temperature for 5 minutes, and read it at 405nm by a microplate reader.

A 0.1% liver homogenate was prepared to measure SOD activity. The reagents were added sequentially according to the instructions, then the samples were incubated at 37°C for 20 min and then read at 450 nm by an enzyme marker.

A 10% liver tissue homogenate was taken according to the instructions of the MDA Kit, heated in 95°C water for 80 min, washed with cold running water for 15 min, and centrifuged at 4°C, 4000 r/min for 15 min. Then, 200 μL of supernatant was read at 532 nm by a microplate reader.

**Measurement of tissue iron content**

A 10% liver tissue homogenate was prepared. Added the reagents in the centrifuge tube according to the instructions, mixed well and centrifuged. Then placed in boiling water for 5 minutes, cooled with running water for 10 min, and centrifuged at 3500r/min for 10 min. In the end, 200 μL of supernatant was read at 520 nm by a microplate reader.

**Measurement of tissue ferrous iron** **(Fe^2+^) content**

Weighted 30-50mg of liver tissue, prepared homogenate according to the mass of tissue: volume of extract=1:5. Centrifuged at 10000g for 10min at 4℃, 200μL of supernatant was taken in a centrifuge tube, 100μL of reagent I was added and mixed well, and then stood at 37℃ for 10min. 100μL of chloroform was added and mixed well, then centrifuged at 12000g for 10min at 24℃. 200μL of the supernatant was taken in a 96-well plate and read at 593nm by a microplate reader. The upper layer of liquid was sucked into a 96-well plate and read at 593nm.
